# Optical source of individual pairs of colour-conjugated photons

**DOI:** 10.1038/s41598-017-11740-w

**Published:** 2017-09-12

**Authors:** Yury Sherkunov, David M. Whittaker, Vladimir I. Fal’ko

**Affiliations:** 1 National Graphene Institute, University of Manchester, Manchester, M13 9PL United Kingdom; 2 Department of Physics and Astronomy, University of Sheffeld, Sheffield, S10 2TN United Kingdom; 3 School of Physics and Astronomy, University of Manchester, Manchester, M13 9PL United Kingdom

## Abstract

We theoretically demonstrate that Kerr nonlinearity in optical circuits can lead to both resonant four-wave mixing and photon blockade, which can be used for high-yield generation of high-fidelity individual photon pairs with conjugated frequencies. We propose an optical circuit, which, in the optimal pulsed-drive regime, would produce photon pairs at the rate up to 5 × 10^5^ *s* ^−1^ (0.5 pairs per pulse) with ${{\boldsymbol{g}}}^{{\bf{(2)}}}{\bf{(0)}}{\boldsymbol{ < }}{{\bf{10}}}^{-{\bf{2}}}$ for one of the conjugated frequencies. We show that such a scheme can be utilised to generate colour-entangled photons.

## Introduction

The use of individual photons^1^ is one of the key elements in the implementation of quantum technologies in communications security^2, 3^ and quantum computation^4^, which stimulated a great progress in designing solid state single-photon sources^5–12^. Single photon generation can be achieved either by the generation of a correlated photon pair in nonlinear media, with detecting one photon of the pair providing the arrival time of the remaining heralded single photon^8, 9, 11, 12^, or by radiative decay of a single quantum emitter, such as quantum dot or diamond colour centre^5–7^, triggered by an optical pulse. Alternatively, a triggered single photon source could be realised with the help of photon blockade^13–15^, where a single photon in a non-linear cavity blocks the transmission of the second one due to strong photon-photon interaction. Significant progress in the realisation of quantum security protocols, *e*.*g*. based on Ekert91 quantum key distribution^16^, has been made using pairs of entangled photons, such as generated by the bi-exciton decay^17^, spontaneous parametric down-conversion (SPDC) in nonlinear crystals^18, 19^, or four-wave mixing (FWM)^11, 12, 20–22^. To guarantee that only a pair of entangled photons is produced, low pumping intensities had to be used in all of the above methods, leading to a low output of photon pairs^9, 10, 18–22^.

Spontaneous FWM is a process that converts two photons from a coherent light source into a pair of photons with up- and down-shifted (conjugated) frequencies. In fibres and waveguides, such a process generates two-photon states, and additional challenge in developing devices suitable for the generation of correlated photon pairs with high fidelity and high efficiency is related to the noise due to the multi-photon generation^1^ associated with the increasing excitation power required for a high output of the device.

**Figure 1:**
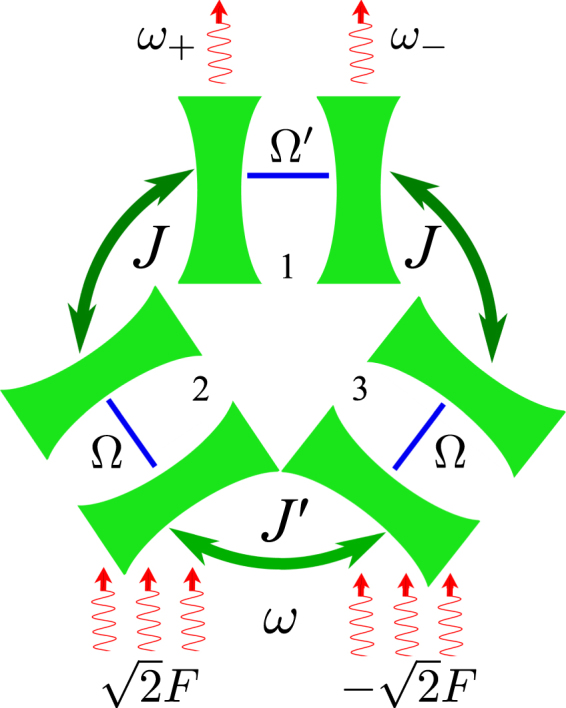
Proposed optical circuit. Coherent pumping with frequency *ω* and amplitudes $\pm \sqrt{2}F$ is applied to single-mode resonators 2 and 3 characterised by frequency Ω. The resonators are coupled with each other by hopping amplitude *J*′ and with resonator 1, which hosts a single photonic mode with frequency Ω′, by hopping amplitude *J*. The system emits correlated photon pairs, with each photon occupying extended conjugated modes *ω*_+_ and *ω*_−_ (see text).

Recent progress in generating strong optical nonlinearities at a few-photon level in the systems, *e*.*g*., where atoms are coupled with a small-mode-volume microcavities^23, 24^, exciton-polariton microcavities^25^, or artificial two-level atoms based on Josephson junctions embedded in microwave resonators^26, 27^, has paved the way for quantum-by-quantum control of light fields. Strong Kerr-type nonlinearity in selectively tuned microcavity-resonators may result in emission of photon pairs in spectrally well-defined modes with tuneable frequencies^28^, with the photon blockade suppressing multiple occupation of conjugated modes^15, 28^. Hence, we propose an optical circuit design depicted in Fig. 1, where non-linear coupling of three photon modes with conjugated frequencies *ω*_±_ and ${\omega }_{0}\approx \frac{1}{2}({\omega }_{+}+{\omega }_{-})$ can be used for the resonant excitation of pure two-photon pairs with optimised high-yield output, followed by the Rabi-type mixing^29^ of the two-photon states |2_0_, 0_+_, 0_−_〉 and 0_0_, 1_+_, 1_−_〉 corresponding to the double occupation of the mode with the frequency *ω*_0_ and single occupation of each of the modes with the frequencies *ω*_±_ respectively, which might be used to produce entangled states of colour-conjugated photon pairs^30^.

The most promising system to address this physics are high-quality toroidal or microrod microcavities, well described by single mode approximation^31, 32^ coupled with an optically dressed atomic gas in electromagnetically-induced transparency regime^13, 33^, in which non-linearity is expected to significantly exceed the losses^31, 33, 34^.

## Model

The proposed circuit, as shown in Fig. 1, can be envisaged as three coupled non-linear cavity resonators, each characterised by a single photonic mode: the resonators 2 and 3 have equal frequencies, Ω, and the resonator 1 has frequency Ω′. The two cavities with equal frequencies are coupled with the cavity 1 by hopping amplitude *J* and by hopping amplitude *J*′ with each other thanks to spatial overlap of the photon modes in the adjacent resonators, as described by the Hamiltonian ($\hslash =c=1$):

1 $$\begin{array}{rcl}{\hat{H}}^{\mathrm{(0)}} & = & {\rm{\Omega }}^{\prime} {\hat{a}}_{1}^{\dagger }{\hat{a}}_{1}+{\rm{\Omega }}\sum _{i=\mathrm{2,}\,3}{\hat{a}}_{i}^{\dagger }{\hat{a}}_{i}+J^{\prime} {\hat{a}}_{2}^{\dagger }{\hat{a}}_{3}\\  &  & +J\sum _{i=\mathrm{2,3}}{\hat{a}}_{1}^{\dagger }{\hat{a}}_{i}+H.c.\equiv \sum _{k=\mathrm{0,}\pm }{\omega }_{k}{\hat{\beta }}_{k}^{\dagger }{\hat{\beta }}_{k},\end{array}$$

with

2 $$\begin{array}{rcl}{\omega }_{0} & = & {\rm{\Omega }}-Jx,\,{\omega }_{\pm }={\rm{\Omega }}+\frac{J}{2}(\delta +x\pm s),\\ s & = & \sqrt{{(\delta -x)}^{2}+8},\end{array}$$

where ${\hat{a}}_{i}$ (${\hat{a}}_{i}^{\dagger }$) are the annihilation (creation) operators of photons in each resonator, frequency detuning, *δ* = (Ω′ − Ω)/*J*, and hopping amplitude mismatch *x* = *J*′/*J*. Energies *ω*_0,±_ correspond to extended eigenmodes of *H* ^(0)^,

3 $$\begin{array}{rcl}{\hat{\beta }}_{0} & = & \frac{1}{\sqrt{2}}({\hat{a}}_{3}-{\hat{a}}_{2}),\\ {\hat{\beta }}_{\pm } & = & \frac{1}{\sqrt{8+{c}_{\pm }^{2}}}[{c}_{\pm }{\hat{a}}_{1}+2({\hat{a}}_{3}+{\hat{a}}_{2})],\\ {c}_{\pm } & = & \delta -x\pm s.\end{array}$$

Resonators 2 and 3 are driven by a coherent pump (at frequency *ω* close *ω*_0_) with the amplitudes $\pm \sqrt{2}F$, which provides coupling to the mode ${\hat{\beta }}_{0}$,

4 $${\hat{H}}^{\mathrm{(1)}}=-F{e}^{i\omega t}{\hat{\beta }}_{0}+H\mathrm{.}c\mathrm{.}$$

Then, we take into account Kerr-type nonlinearity, which, for simplicity, will have the same strength, $u\ll J$, on each cavity. It can be described by a Bose-Hubbard model^13^

5 $$\begin{array}{ccc}{\hat{H}}^{(2)} & = & u\sum _{i}{\hat{a}}_{i}^{\dagger }{\hat{a}}_{i}^{\dagger }{\hat{a}}_{i}{\hat{a}}_{i}\\  & = & \kappa ({\hat{\beta }}_{+}^{\dagger }{\hat{\beta }}_{-}^{\dagger }{\hat{\beta }}_{0}^{2}+H.c.)+\sum _{k,{k}^{{\rm{^{\prime} }}}}{\alpha }_{k{k}^{{\rm{^{\prime} }}}}{\hat{\beta }}_{k}^{\dagger }{\hat{\beta }}_{{k}^{{\rm{^{\prime} }}}}^{\dagger }{\hat{\beta }}_{{k}^{{\rm{^{\prime} }}}}{\hat{\beta }}_{k}+\delta \hat{H},\end{array}$$

where

6 $$\begin{array}{ccc}\kappa  & = & \frac{\sqrt{2}u}{s},\,{\alpha }_{00}=\frac{\kappa s}{2\sqrt{2}},\,{\alpha }_{\pm \mp }=\frac{6\kappa }{\sqrt{2}s},\\ {\alpha }_{\pm 0} & = & {\alpha }_{0\pm }=\frac{\kappa }{2\sqrt{2}}[s\mp (\delta -x)],\\ {\alpha }_{\pm \pm } & = & \frac{\kappa }{4\sqrt{2}s}\mathrm{[3}{s}^{2}-12\pm (\delta -x)s\mathrm{].}\end{array}$$

The second line in ${\hat{H}}^{\mathrm{(2)}}$ represents interaction between the extended modes, *β*_0,±_. Here, the first term describes resonant four-wave mixing of two *ω*_0_ photons with the pair of photons at the conjugated frequencies, *ω*_±_ described by the FWM coupling constant *κ*. The second term produces occupancy-dependent shifts in the photon frequencies. The rest of the terms generated by the canonical transformation from the single-cavity to the extended modes are combined into a perturbation $\delta \hat{H}$; under conditions which will be identified below, these terms are non-resonant for the production process of the photon pairs with conjugated frequencies, hence, they give only a small contribution. However, in our numerical analysis below we take this contribution into account.

Now let us consider the two photon states in the system corresponding to *n*_0_ + 2 photons in the mode *ω*_0_ and *n*_+_ (*n*_−_) photons in the mode *ω*_+_ (*ω*_−_)

7 $$|{n}_{0}+2,\,{n}_{+},\,{n}_{-}\rangle =\frac{{({\hat{\beta }}_{0}^{\dagger })}^{{n}_{0}+2}{({\hat{\beta }}_{+}^{\dagger })}^{{n}_{+}}{({\hat{\beta }}_{-}^{\dagger })}^{{n}_{-}}}{\sqrt{({n}_{0}+2)!{n}_{+}!{n}_{-}!}}|0\rangle ,$$

and *n*_0_ photons in the mode *ω*_0_ and *n*_+_ + 1 (*n*_−_ + 1) photons in the mode *ω*_+_ (*ω*_−_)

8 $$|{n}_{0},{n}_{+}+1,{n}_{-}+1\rangle =\frac{{({\hat{\beta }}_{0}^{\dagger })}^{{n}_{0}}{({\hat{\beta }}_{+}^{\dagger })}^{{n}_{+}+1}{({\hat{\beta }}_{-}^{\dagger })}^{{n}_{-}+1}}{\sqrt{{n}_{0}!({n}_{+}+1)!({n}_{-}+1)!}}|0\rangle .$$

The probability of FWM between the states given by Eqs (7) and (8) peaks when the resonance condition

9 $$E({n}_{0}+2,\,{n}_{+},\,{n}_{-})=E({n}_{0},\,{n}_{+}+1,\,{n}_{-}+1)$$

is satisfied, where *E*(*n*_0_, *n*_+_, *n*_−_) is the diagonal matrix element of the Hamiltonian $\hat{H}={\hat{H}}^{(0)}+{\hat{H}}^{(1)}+{\hat{H}}^{(2)}$, $E({n}_{0},{n}_{+},{n}_{-})=\langle {n}_{0},{n}_{+},{n}_{-}|\hat{H}|{n}_{0},{n}_{+},{n}_{-}\rangle$ ^35^, making the states (7) and (8) degenerate. In this case, generation of pairs of *ω*_±_-photons is promoted by the resonance conditions for converting them from the pairs of pumped photons. Condition (9) can be obtained by tuning the frequency detuning *δ* in $\hat{H}$ to the value

10 $$\delta =-3x+\frac{\kappa }{2J}[\frac{4{x}^{2}-1}{\sqrt{1+2{x}^{2}}}\mathrm{(2}+{n}_{+}+{n}_{-})+10\sqrt{2}x({n}_{+}-{n}_{-})],$$

which can be easily seen by substituting Eqs (1), (2), (5) and (6) into Eq. (9). The latter expression was obtained by neglecting terms $\delta \hat{H}$ in ${\hat{H}}^{\mathrm{(2)}}$. This indicates that the resonance conditions for the states involving different occupation numbers *n*_±_ can be separated, whereas the resonance conditions for the processes |*n*_0_ + 2, 0_+_, 0_−_〉 ⇔ |*n*_0_, 1_+_, 1_−_〉 generating a single pair of photons at conjugated frequencies *ω*_±_,

11 $$\delta =-3x+\frac{4{x}^{2}-1}{4{x}^{2}+2}\frac{u}{J},$$

is the same for all values of *n*_0_. This condition sets the values of the parameters in Eqs (1) and (5) to

12 $$\begin{array}{rcl}{\omega }_{0} & = & {\rm{\Omega }}-Jx;\,\kappa \approx \frac{u}{2\sqrt{1+2{x}^{2}}};\\ {\omega }_{\pm } & = & {\rm{\Omega }}-(x\mp \sqrt{2}\sqrt{1+2{x}^{2}})J+\frac{4{x}^{2}-1}{4{x}^{2}+2}(\sqrt{1+2{x}^{2}}\mp \sqrt{2}x)\kappa ;\\ {\alpha }_{00} & = & \sqrt{1+2{x}^{2}}\kappa ;\,{\alpha }_{\pm \pm }=\frac{3+12{x}^{2}\mp 2x\sqrt{2+4{x}^{2}}}{4\sqrt{1+2{x}^{2}}}\kappa ;\\ {\alpha }_{\pm 0} & = & {\alpha }_{0\pm }=[\sqrt{1+2{x}^{2}}\pm \sqrt{2}x]\kappa ;\,{\alpha }_{\pm \mp }=\frac{3\kappa }{2\sqrt{1+2{x}^{2}}}\mathrm{.}\end{array}$$

## Results

### Preliminary analysis

To get an idea about spectral properties of this optical circuit under the resonance conditions (11)–(12), we neglect the non-resonant term $\delta \hat{H}$ in *H* ^(2)^ (see discussion below Eq. (6)) and diagonalise

13 $$\hat{H}={\hat{H}}^{(0)}+{\hat{H}}^{(2)}$$

in the basis of the Fock states with total photon number in the system not exceeding *N* = 4. This approximation of the Hilbert space is justified in the case of weak pumping we consider in this article. Then, the spectrum of *N* −photon states is:

14 $$\begin{array}{rcl}E(0) & = & 0,\,\,E(1)={\omega }_{0},\\ |0\rangle  & = & |{0}_{0},{0}_{+},{0}_{-}\rangle ,\,\,|1\rangle =|{1}_{0},{0}_{+},{0}_{-}\rangle ;\\ E(2\alpha ) & \approx  & 2{\omega }_{0}+(\sqrt{2+4{x}^{2}}\pm 1)\sqrt{2}\kappa ,\\ |2\alpha \rangle  & = & \frac{1}{\sqrt{2}}(|{2}_{0},{0}_{+},{0}_{-}\rangle \pm |{0}_{0},{1}_{+},{1}_{-}\rangle );\\ E(3\alpha ) & \approx  & 3{\omega }_{0}+(\sqrt{6+12{x}^{2}}\pm 1)\sqrt{6}\kappa ,\\ |3\alpha \rangle  & = & \frac{1}{\sqrt{2}}(|{3}_{0},{0}_{+},{0}_{-}\rangle \pm |{1}_{0},{1}_{+},{1}_{-}\rangle ).\end{array}$$

**Figure 2:**
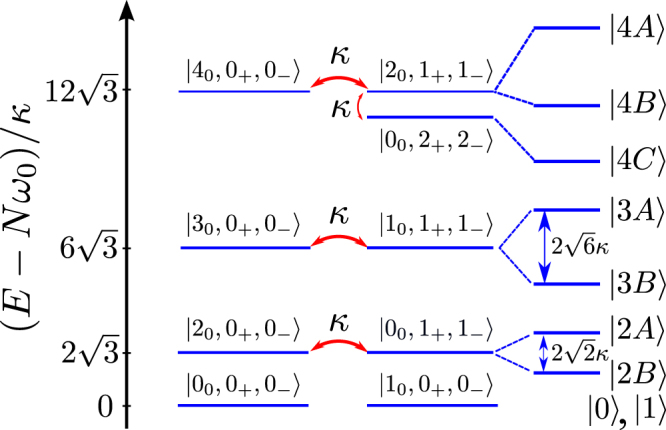
Energy spectrum of the system. Resonant mixing and splitting of *N*-photon states |*n*_0_, *n*_+_, *n*_−_〉 (*N* = *n*_0_ + *n*_+_  + *n*_−_) coupled by the FWM described by the truncated Hamiltonian ${\hat{H}}^{\mathrm{(2)}}$. The frequency detuning *δ* = (Ω′ − Ω)/*J* is chosen in such a way that the energy levels corresponding to the states |*n*_0_ + 2, 0_+_, 0_−_〉 and |*n*_0_, 1_+_, 1_−_〉 are in resonance, while the energy levels of the states with multiple occupation of the modes *ω*_+_ and *ω*_−_ are red-shifted. Energies of relevant states with *N* = 0, 1, 2, 3 photons are given in Eq. (14).

Here, upper/lower signs correspond to the states *A*/*B* among |*Nα*〉 (*N* = 0, 1, 2, 3; *α* = *A*, *B*) marked in Fig. 2. In a pumped system, absorption of photons by the system would be resonantly favoured when *N* incident photons have the same energy as *N* photons in the cavity, *Nω* = *E*(*Nα*). Thus, Eq. (14) and Fig. 2 provide information about resonant pumping frequencies required to excite corresponding photon states |*Nα*〉 in the system. Note that such multi-photon resonances can also be found in weakly dissipating systems.

### Numerical analysis

Now we turn our attention to a more realistic system, with coherent pumping with amplitudes $\pm \sqrt{2}F$ and frequency *ω* applied to resonators 2 and 3 (see Fig. 1), and described by the Hamiltonian

15 $$\hat{H}={\hat{H}}^{(0)}+{\hat{H}}^{(1)}+{\hat{H}}^{(2)}.$$

We also take into account photon losses in the system due to finite mirror transmittivity quantified by frequency-independent decay rate *γ*. The evolution of such a system can be described using the master equation,

16 $$\frac{\partial \hat{\rho }}{\partial t}=-i[\hat{H},\hat{\rho }]+\gamma \sum _{i=0,1,2}(2{\hat{\beta }}_{i}\hat{\rho }{\hat{\beta }}_{i}^{\dagger }-{\hat{\beta }}_{i}^{\dagger }{\hat{\beta }}_{i}\hat{\rho }-\hat{\rho }{\hat{\beta }}_{i}^{\dagger }{\hat{\beta }}_{i}),$$

for the density matrix, *ρ*, which we write in a Fock basis

17 $$\begin{array}{rcl}\hat{\rho } & = & \sum \rho ({m}_{0},{m}_{+},{m}_{-};{n}_{0},{n}_{+},{n}_{-})\\  &  & |{m}_{0},{m}_{+},{m}_{-}\rangle \langle {n}_{0},{n}_{+},{n}_{-}|.\end{array}$$

By solving this master equation numerically using the basis that includes states with up to *N*_*max*_ ≤ 10 photons in each mode, we calculate the occupation numbers ${N}_{i}={\rm{Tr}}[{\hat{\beta }}_{i}^{\dagger }{\hat{\beta }}_{i}\hat{\rho }]$, zero time-delay pair correlation functions for each mode *ω*_0,±_, ${g}_{i}^{\mathrm{(2)}}={\rm{Tr}}[({\hat{\beta }}_{i}^{\dagger }{)}^{2}{\hat{\beta }}_{i}^{2}\rho ]/{N}_{i}^{2}$ and the probabilities *P*(2_0_) and *P*(1_+_, 1_−_) to find a photon pair in the mode *ω*_0_ or the conjugated modes, respectively. We checked the consistency of our calculations by converging the results upon increasing *N*_*max*_ and by comparing the results of modelling where we include and neglect the interaction terms $\delta \hat{H}$.

**Figure 3:**
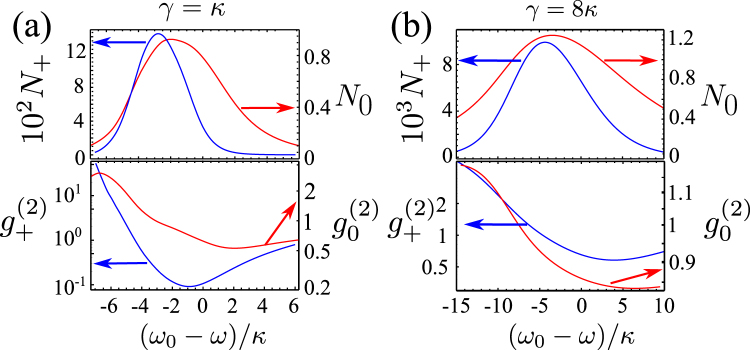
The system with large losses. Steady state under continuous pumping, as a function of pump frequency *ω*. (**a**) *γ* = *κ*, *F* = 2*κ*; (**b**) *γ* = 8*κ*, *F* = 10*κ*.

**Figure 4:**
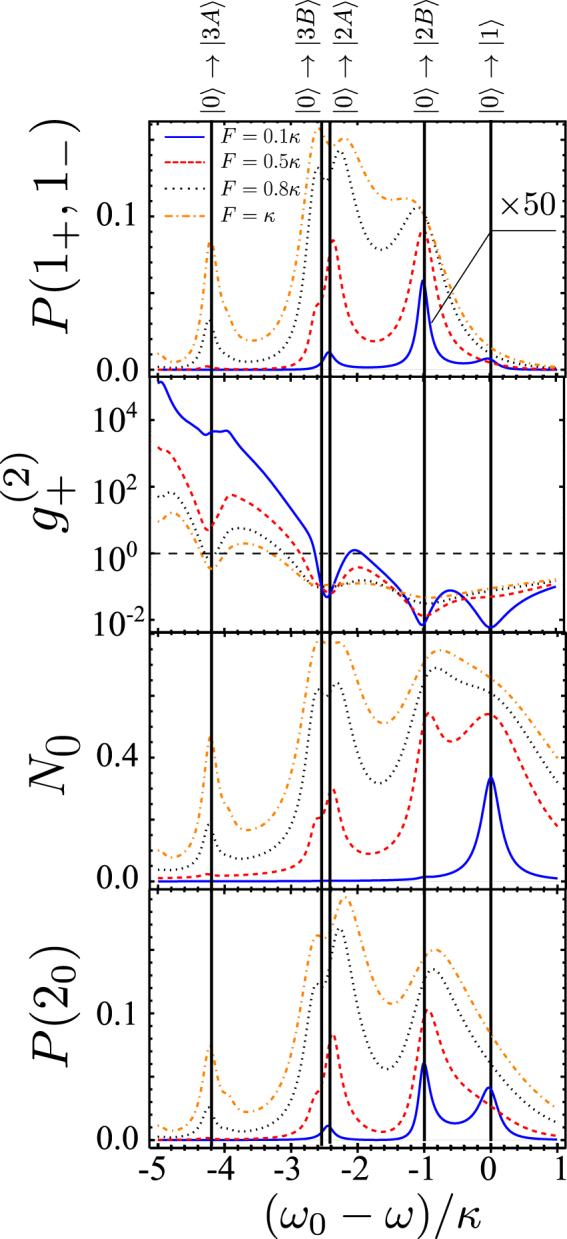
The system with small losses. Dependence of steady state values of *P*(1_+_, 1_−_), ${g}_{+}^{(2)}$, *N*_0_ and *P*(2_0_) on the pump frequency *ω* for *γ* = 0.1*κ* and various amplitudes of the pump. Relevant resonance conditions correspond to multi-photon transitions sketched in Fig. 2.

First, we solved Eq. (16) for a circuit with *J*′ = *J* (*x* = 1) and *γ* = *κ* or *γ* = 8*κ* (which is typical for GaAs polaritonic microcavities^25^), with weak anti-bunching in the low-flux of *ω*_±_ photons demonstrated by ${g}_{+}^{(2)}$ shown in Fig. 3. In contrast, for the resonant conditions set for *J*′ = *J* (*x* = 1) and with *γ* = 0.1*κ* in the system continuously pumped with amplitude *F*, we find a much higher efficiency of production of pure two-photon states. The numerically found steady-state solutions of Eq. (16) display resonances corresponding to the transitions in the spectrum in Fig. 2. This is illustrated in Fig. 4 by the pump-frequency *ω* dependence of probabilities *P*(1_+_, 1_−_) and *P*(2_0_) to find one *ω*_±_ pair or two *ω*_0_ photons in the circuit, occupation numbers for the excited *ω*_0_ photons, and the two-photon correlation function ${g}_{+}^{(2)}$. For $F\ll \kappa$, *P*(2_0_) and *P*(1_+_, 1_−_) have pronounced resonances at 2*ω* = *E*(2*A*) and 2*ω* = *E*(2*B*), corresponding to the two-photon transitions |0〉 → |2*A*〉 and |0〉 → |2*B*〉. Resonant excitation of individual *ω*_±_ pairs is also reflected by the dips in ${g}_{+}^{(2)}$. For larger *F*, we identify additional resonances in the vicinity of 3*ω* = *E*(3*A*) and 3*ω* = *E*(3*B*), corresponding to the three-photon transitions |0〉 → |3*A*〉 and |0〉 → |3*B*〉. Note that, at a larger *F*, the pump induces shifts in the resonance conditions to create multi-photon states, Eq. (14), and the maxima, *e*.*g*. in *N*_0_ shown in Fig. 4, are additionally shifted by power-dependent broadening of |0〉 → |1〉 resonance. These pump-induced shifts can be estimated under the assumptions that $F\ll \kappa$, $\gamma \ll F$, and $\gamma \ll \kappa$. Solving Eq. (16) analytically in the Fock basis truncated at the total number of photons *N* = 3, we find

18 $$\begin{array}{rcl}E(2A) & \to  & E(2A)-1.34{F}^{2}/\kappa ,\\ E(2B) & \to  & E(2B)-0.23{F}^{2}/\kappa ,\\ E(3A) & \to  & E(3A)+0.32{F}^{2}/\kappa ,\\ E(3B) & \to  & E(3B)+2.03{F}^{2}/\kappa ,\end{array}$$

which is in good qualitative agreement with the numerical results shown in Fig. 4.

**Figure 5:**
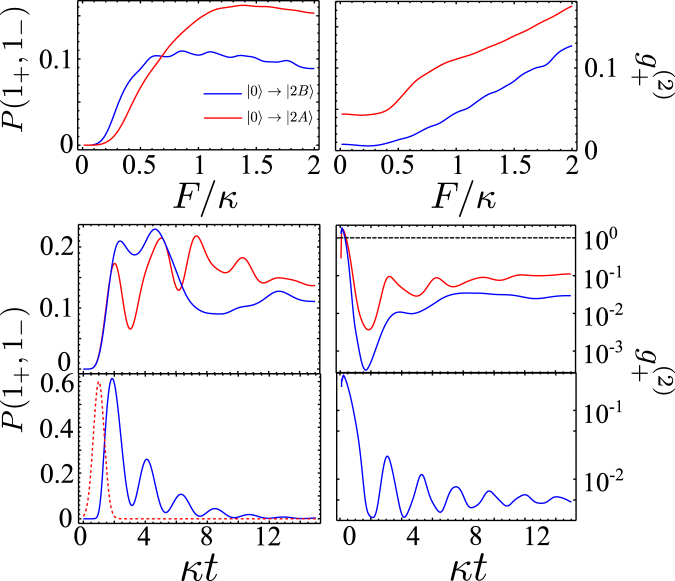
Steady state parameters and time evolution of the system with small losses. Steady-state parameters computed in the system with *γ* = 0.1*κ* under continuous pumping as a function of pumping amplitude *F* for resonance frequencies of |0〉 → |2*A*〉 and |0〉 → |2*B*〉 transitions marked on Fig. 5 (top). Time evolution of pumped circuit, following the switching-on of the pump *F* = 0.8*κ* (middle). Rabi-type oscillations of two-photon states in a circuit pumped by a Gaussian pulse (dashed line) with parameters indicated in Fig. 6 (*τ* = 0.5/*κ* and *F*_0_ = 2.6*κ*) (bottom).

As the pumping amplitude grows, the two-photon resonances |0〉 → |2*A*〉 and |0〉 → |2*B*〉 become more pronounced, accompanied by increasing *P*(1_+_, 1_−_). However, as shown in the two top panels of Fig. 5, the joint probability *P*(1_+_, 1_−_) demonstrates saturation at $F \sim \kappa$ for the circuit pumped at resonance frequencies of |0〉 → |2*A*〉 and |0〉 → |2*B*〉 transitions. At the same time ${g}_{+}^{(2)}$ increases signifying the pollution of photon pairs at conjugated frequencies with individual *ω*_±_ photons. This suggests that a mere increase of pumping does not improve the output of correlated photon pairs.

## Discussion

**Figure 6:**
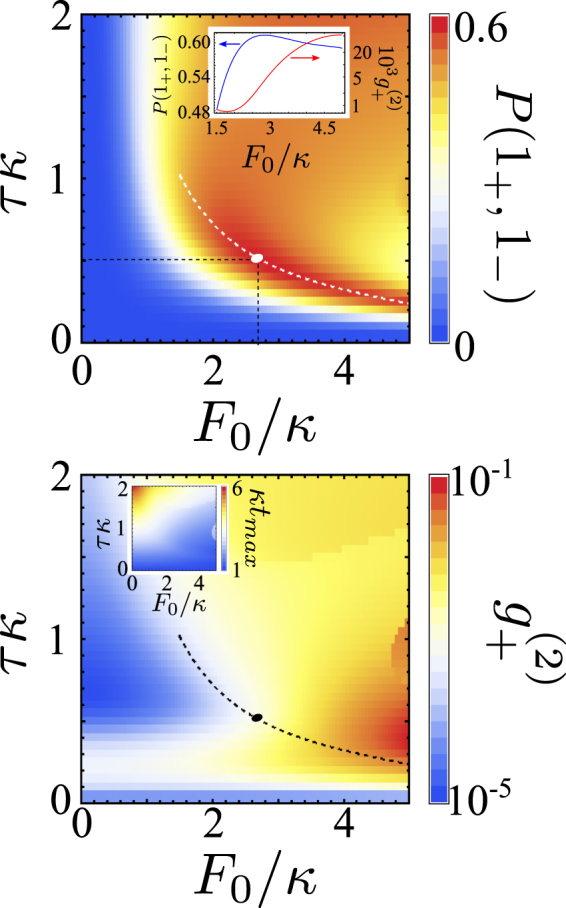
Parameters of the system pumped by a Gaussian pulse. *P*(1_+_, 1_−_) (top) and ${g}_{+}^{(2)}$ (bottom) at *t* = *t*_*max*_ for the system pumped by a Gaussian pulse with amplitude $F={F}_{0}{e}^{-{(t-2\tau )}^{2}/{\tau }^{2}}\theta (t)$. White (black) dot marks a favourable choice of *τ* and *F*_0_ used in Fig. 5. Insets: (top) correlated photon pair output and ${g}_{+}^{(2)}$ along the line of maximal *P*(1_+_, 1_−_) at *t* = *t*_*max*_ and (bottom) parametric dependence of *t*_*max*_ *κ*.

The insight into how one can increase the output of individual photon pairs comes from the pronounced beatings in the temporal evolution of *P*(1_+_, 1_−_) and ${g}_{+}^{(2)}$, which follow switching-on of the excitation source *F* = *θ*(*t*) × const (*θ* is the Heaviside function), Fig. 5. These beatings are the results of two-photon Rabi oscillations^29^, generated by resonance mixing of |*n*_0_ + 2, 0_+_, 0_−_〉 ⇔ |*n*_0_, 1_+_, 1_−_〉 states. Hence, we suggest to implement pulsed excitations, harvesting photon pairs within optimally chosen delay-time windows. Note that, for the FWM coupling constant $\kappa \ll J$, the period, $\pi /\sqrt{2}\kappa$, of Rabi oscillations |2, 0_+_, 0_−_〉 ⇔ |0, 1_+_, 1_−_〉, is long enough for harvesting correlated photon pairs at the time interval around the optimal delay *t*_*max*_ at the maximum of *P*(1_+_, 1_−_), without undermining their spectral identity. Hence, we identify time intervals of the maximal probability to find a high-fidelity conjugated photon pair (in those intervals, *N*_+_ ≈ *P*(1_+_, 1_−_)). An example of time-dependent *P*(1_+_, 1_−_) and ${g}_{+}^{(2)}$, produced by a Gaussian pulse of duration *τ*, is shown in the bottom panels in Fig. 5, and in Fig. 6 we show the dependence of the size of the maximum output *P*(1_+_, 1_−_) and ${g}_{+}^{(2)}$, at *t*_*max*_. The optimal choice of the duration and amplitude of the pulse offers a high yield, $P({1}_{+},{1}_{-}) \sim 0.5$ of an almost pure two-photon state with ${g}_{+}^{(2)}(0)\approx {10}^{-2}$.

To achieve the desirable regime of $\kappa /\gamma \gg 1$, one needs to use materials with a large non-linearity and cavities with a high quality factor, *Q*. Depending on the operational frequency range, these may be $Q \sim {10}^{9}$ superconducting microwave resonators^26, 27^, coupled with superconducting qbits to provide strong Kerr-nonlinearity for microwave frequencies, or trapped atoms in the electromagnetically-induces-transparency regime^13, 33^ resonantly coupled to $Q \sim {10}^{7-8}$ toroidal^31^ or microrod^32^ microcavities to provide strong non-linearity for visible or infrared frequencies. In the latter systems^31, 33, 34^, non-linearity can reach $\kappa  \sim 1.25\times {10}^{7}{s}^{-1}$, with $\gamma  \sim {10}^{6}{s}^{-1}$, and the optimised pulses with repetition rate *γ* would produce pairs of colour-conjugated photons with ${g}_{s}^{\mathrm{(2)}}\mathrm{(0)} \sim {10}^{-2}$ at the rate of up to 0.5 × 10^6^ *s* ^−1^ or 0.5 photon pairs per an excitation pulse, higher than that achievable for the parametric down-conversion process. Indeed, the state at the output of the down-conversion process in a non-linear crystal generated by a laser pulse is a two-mode squeezed state^36^

19 $$|{{\rm{\Psi }}}_{SPDC}\rangle =\sqrt{1-|\mu {|}^{2}}\sum _{n=0}^{\infty }{\mu }^{n}|n{\rangle }_{s}|n{\rangle }_{i},$$

where |*n*〉_*s*_ (|*n*〉_*i*_) is an *n*-photon Fock state of the signal (idler) mode and *μ* is the squeezing parameter, which depends on the parameters of the laser pulse incident on the crystal. The purity of the source is characterised by the normalised zero-time-delay idler-triggered second-order autocorrelation function of the signal mode^37^

20 $${g}_{s}^{(2)}(0)=\frac{{\langle {({\hat{\alpha }}_{s}^{\dagger })}^{2}{\hat{\alpha }}_{s}^{2}\rangle }_{i}}{{\langle ({\hat{\alpha }}_{s}^{\dagger }){\hat{\alpha }}_{s}\rangle }_{i}^{2}},$$

where postselection is taken into account by the projection of the idler states ${\langle \hat{x}\rangle }_{i}=\langle {\hat{\alpha }}_{i}^{\dagger }\hat{x}{\hat{\alpha }}_{i}\rangle /\sqrt{\langle {\hat{\alpha }}_{i}^{\dagger }{\hat{\alpha }}_{i}\rangle }$. Here, ${\hat{\alpha }}_{i}$ (${\hat{\alpha }}_{s}$) is the annihilation operator of a photon in the idler (signal) mode. Thus, for the state generated by the SPDC process (19) and $\mu \ll 1$, one finds ${g}_{s}^{\mathrm{(2)}}\mathrm{(0)}$ ≈ 4*μ*(1 − 2*μ* ^2^). At the same time, the average number of photon pairs per pulse can be found as^37^ $\langle {n}_{s}\rangle ={\langle {\hat{\alpha }}_{s}^{\dagger }{\hat{\alpha }}_{s}\rangle }_{i}\approx \mu \mathrm{(1}+5{\mu }^{2}\mathrm{/2)}$ leading to 〈*n*_*s*_〉 ≈ 2.5 × 10^−3^ pairs per pulse with purity *g*_*s*_^(2)^(0) = 10^−2^. Thus, the proposed setup would produce correlated photon pairs with the yield 200 times larger then a conventional SPDC source provided the purity is *g*_*s*_^(2)^(0) = 10^−2^. The purity and yield of the proposed source of correlated photons would also be better than what has been predicted theoretically^38^ and achieved experimentally^10^ for the parametric down-conversion process in the cavity-waveguide based systems. Indeed, Pomarico *et al*.^38^ have theoretically demonstrated that the optimal production rate per pump power in narrow-band integrated cavity-waveguide systems based on the parametric down-conversion is 4.8 × 10^7^(*smW*)^−1^, which, with experimentally available powers not exceeding 2.2 *μW* (for photon pair generation with ${g}_{s}^{\mathrm{(2)}}\mathrm{(0)} \sim 0.1$)^10^, limits the production rate to 10^5^ *s* ^−1^.

Finally, the proposed non-linear optical circuit can be used as a colour-entangled photon source^30^ by connecting one waveguide L to resonator 1 and another waveguide R equally coupled to both resonators 2 and 3 used for the excitation pulse. The escape of the two-photon state |1_+_, 1_−_〉 into the waveguides L/R, with couplings ~*γ*, would deliver signals to the recipients at the L and R ends,

21 $$\begin{array}{rcl}|{1}_{+},{1}_{-}\rangle  & \to  & [\frac{1}{2}-\frac{x}{\sqrt{2}\sqrt{1+2{x}^{2}}}]|{L}_{+},{R}_{-}\rangle \\  &  & -[\frac{1}{2}+\frac{x}{\sqrt{2}\sqrt{1+2{x}^{2}}}]|{R}_{+},{L}_{-}\rangle \\  &  & +\frac{1}{2\sqrt{1+2{x}^{2}}}[|{R}_{+},{R}_{-}\rangle -|{L}_{+},{L}_{-}\rangle ],\end{array}$$

who would detect arrival of photons, distinguishing their colour. Projecting the wave function Eq. (21) onto the subspace with one photon at L and one at R, recipients L and R would be able to use the colour-entangled photon pairs similarly to what was suggested for the polarisation-entangled photon pairs^1, 17, 18^. Then, optimal output of the colour-entangled states would be achieved in a circuit with hopping amplitude mismatch *x* → 0 ($J^{\prime} \ll J$, corresponding to a simple linear chain of three cavity-resonators with ${\omega }_{\pm }\approx {\omega }_{0}\pm \sqrt{2}J$, well separated from both *ω*_0_ mode and the pumping field). Indeed, for a typical value *J* = 0.5 meV achievable for a system of coupled toroidal or microrod microcavities^39^, the frequency separation is of order ${\omega }_{\pm }-{\omega }_{0} \sim {10}^{12}{s}^{-1}$, which significantly exceeds the value of available FWM coupling constant $\kappa  \sim 1.25\times {10}^{7}{s}^{-1}$. In this case, the first two lines of Eq. (21) would describe nothing but a Bell pair.
